# Pikeperch muscle tissues: a comparative study of structure, enzymes, genes, and proteins in wild and farmed fish

**DOI:** 10.1007/s10695-024-01354-1

**Published:** 2024-05-11

**Authors:** Katrin Tönißen, George P. Franz, Elke Albrecht, Philipp Lutze, Ralf Bochert, Bianka Grunow

**Affiliations:** 1 Fish Growth Physiology Workgroup, Research Institute for Farm Animal Biology (FBN), Wilhelm-Stahl-Allee 2, 18196 Dummerstorf, Germany; 2 Working Group Muscle-Fat Crosstalk, Research Institute for Farm Animal Biology (FBN), Wilhelm-Stahl-Allee 2, 18196 Dummerstorf, Germany; 3 Mecklenburg-Vorpommern Research Centre for Agriculture and Fisheries (LFA MV), Institute of Fisheries, Research Station Aquaculture, Born, Germany

**Keywords:** Percidae, Muscle fibre structure, Enzyme activity, Protein abundance, Gene expression

## Abstract

Pikeperch (*Sander lucioperca*) is a freshwater species and an internationally highly demanded fish in aquaculture. Despite intensive research efforts on this species, fundamental knowledge of skeletal muscle biology and structural characteristics is missing. Therefore, we conducted a comprehensive analysis of skeletal muscle parameters in adult pikeperch from two different origins, wild-caught specimens from a lake and those reared in a recirculating aquaculture system. The analyses comprised the biochemical characteristics (nucleic acid, protein content), enzyme activities (creatine kinase, lactate dehydrogenase, NADP-dependent isocitrate dehydrogenase), muscle-specific gene and protein expression (related to myofibre formation, regeneration and permanent growth, muscle structure), and muscle fibre structure. The findings reveal distinct differences between the skeletal muscle of wild and farmed pikeperch. Specifically, nucleic acid content, enzyme activity, and protein expression varied significantly. The higher enzyme activity observed in wild pikeperch suggests greater metabolically activity in their muscles. Conversely, farmed pikeperch indicated a potential for pronounced muscle growth. As the data on pikeperch skeletal muscle characteristics is sparse, the purpose of our study is to gain fundamental insights into the characteristics of adult pikeperch muscle. The presented data serve as a foundation for further research on percids’ muscle biology and have the potential to contribute to advancements and adaptations in aquaculture practices.

**Supplementary Information:**

The online version contains supplementary material available at 10.1007/s10695-024-01354-1.

## Introduction

Muscle is a heterogeneous tissue that contains various cell types, including myocytes, adipocytes, and fibroblasts. In fishes, the development of muscle tissue (myogenesis) can occur either through the growth of cells (hypertrophy) or through the formation of new cells (hyperplasia) (Johnston et al. 2011). The complex cascade of myogenesis begins with the activation, proliferation, differentiation, and maturation of myogenic precursor cells, all of which are regulated by myogenic regulatory factors (MRFs). The interplay between genetic regulation and the signalling pathways of MRFs leads to the differentiation of myoblasts (Koganti et al. 2020) with subsequent differentiation of myoblasts into myotubes and finally to the formation of multinucleated myofibres.

In the trunk muscles of adult teleost, the white muscle comprises 90–95% of total muscle mass and is responsible for short bursts of rapid swimming (Kiessling et al. 2006). The slow red muscle allows the fish to swim slowly and steadily, characterised by a slow contraction speed and aerobic substrates used by its muscle fibres (Johnston 1981). The pink or intermediate muscle, if present, occupies an anatomical and physiological position between the red and white muscle and occurs in varying amounts (Mascarello et al. 1986, Rowlerson et al. 1985, Sänger and Stoiber 2001). Yada et al. (2001) determined the pink muscle fibre in 27 fish species and showed significant variations in their number and surface area. The cellular mechanisms of myogenesis in previously studied fish species are diverse and differ from those of mammals. Despite their high degree of conservation in genetic regulation, as noted by Rossi and Messina (2014) and Koganti et al. (2020), the effects of domestication on myogenesis and muscle tissue are still unexplored. Comparative studies between wild and farmed fish have consistently demonstrated that the fillet of wild fish was perceived to have a better taste and flavour (for Atlantic salmon, *Salmon salar*; Johnston et al. 2006, gilthead sea bream, *Sparus aurata*; Grigorakis 2007, and European sea bass, *Dicentrarchus labrax*; Grigorakis 2007, Fuentes et al. 2010). A complete explanation for these differences is not yet present, but potential factors contributing to these differences may be found in muscle cellularity and collagen content, as well as the general life history of the fish (Periago et al. 2005). The complex interplay of numerous biotic and abiotic factors influences muscle development and thus the properties of muscle tissue (Totland et al. 1987, Johnston 1999, Salem et al. 2013, Videler 2011, Zhao et al. 2018).

The understanding of this dynamic process is supported by the characterisation of energy metabolism using enzyme activities extensively studied in fish muscle from the 1960s to 1980s (e.g., Bouck and Ball 1968, Frankel 1980, Lim et al. 1975, Wilson et al. 1973). Other researchers studied the fibre-specific amounts of nuclei in different teleost species (Koumans et al. 1991, 1994; Priester et al. 2011). As no comparable studies have been conducted on pikeperch (*Sander lucioperca*), there is a notable lack of information on its muscle characteristics and the effects of genetic and environmental factors on its skeletal muscle tissue (Islam 2006; Policar et al. 2016; Prychepa et al. 2018; Komolka et al. 2020; Tönißen et al. 2022).

Pikeperch is an internationally highly demanded and high-value food fish that is native to the fresh and brackish waters of Eastern Europe as far as the Caspian Sea. This coincides with the most important producing countries the Czech Republic, Denmark, Hungary, Romania, Tunisia, and Ukraine (FAO, 2023). To gain insights into the muscle biology and physiology of pikeperch, a thorough investigation of muscle development is essential.

The objective of the current study was to investigate the biochemical, genetic, and histological parameters of skeletal muscle in wild and farmed pikeperch. This research aims to enhance our understanding of the biology of pikeperch muscle tissue and shed light on the effects of domestication or specific husbandry systems like recirculating aquaculture systems (RAS).

## Materials and methods

### Animal rearing and sampling

Wild pikeperch were captured from a fishery at Lake Hohen Sprenz in Mecklenburg-Vorpommern, Germany, and farmed specimens were obtained from the Mecklenburg-Vorpommern Research Centre for Agriculture and Fisheries in Born, Germany. All animals were comparable in total length and size, so no significant differences between these two groups were present. The aquaculture pikeperch had a total length of 48.3 ± 0.8 cm, a circumference of 22.5 ± 0.5 cm, and total weight of 896.4 ± 46.9 g. The wild animals obtained from the freshwater lake Hohen Sprenz indicated a total length of 45.7 ± 1.3 cm, a circumference of 21.8 ± 0.4 cm, and a total weight of 710.7 ± 32.1 g.

The ten wild pikeperch were collected in spring (average water temperature 14.7 ± 1.6 °C), matching the sampling period for RAS-farmed pikeperch. Age determination was based on the total length, which was comparable to the size of cultured pikeperch. Seven farmed pikeperch were obtained for the experiment, originating from a batch of 24-month-old animals. The RAS system was composed of ten round tanks, each 3 m^3^ in size. Water temperature was maintained at 20.9 ± 1.8 °C, and 24-h lighting was used. RAS-farmed animals were fed a commercial diet (Coppens Supreme-10, 4.5 mm pellet size, crude protein (CP) 49%, crude fat (CF) 10%, ash 9.4%, fibre 1.8%, digestible energy (DE) 15.5 MJ) with a feeding rate of 1.0% body weight. For analysis, all fish were transported to the Research Institute for Farm Animal Biology (FBN) in Dummerstorf, Germany, using a transport tank with an additional oxygen supply. To account for animal welfare and to reduce stress, transport was only performed in groups of a maximum five specimens (density of 35 kg/m^3^). Sampling and processing of each sampling group were carried out within 1 to 2 days.

**Fig. 1:**
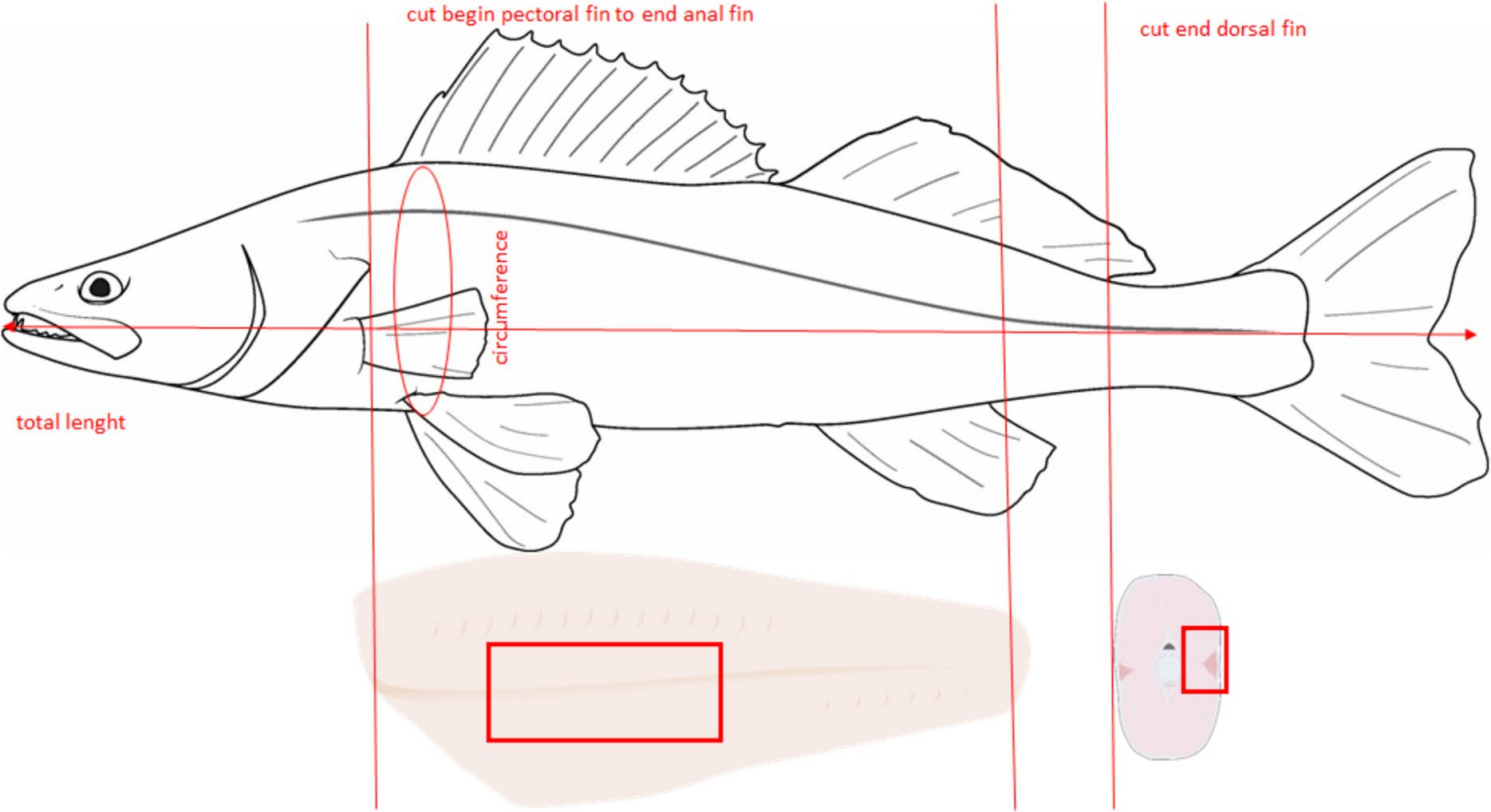
Schematic of the morphometric characters and sampling for the biochemical, expression, and histological analyses on pikeperch

This study followed international, national, and institutional guidelines for the treatment and sacrifice of animals and complied with relevant legislation. Fish were euthanised prior to sampling according to the Animal Welfare Directive 2010/63/EU and the German Animal Welfare (Act TierSchG § 4(3)) by a beat on the head and were directly killed by a stab to the heart and severing of the spinal cord behind the head. The sampling procedure was previously described in detail by Komolka et al. (2020) and Grunow et al. (2021). For biochemical and expression analysis, samples of dorsal and ventral white muscle along the horizontal septum were taken from each specimen (Fig. 1), snap-frozen in liquid nitrogen, and stored at − 80 °C. For histological analysis, < 1-cm-thick caudal peduncle sections were cut (Fig. 1), shock frozen in liquid nitrogen in combination with precooled isopentane and stored at − 80 °C.

### Biochemical analyses

The DNA content of muscle tissue was measured using Hoechst 33,258 (Sigma-Aldrich) and quantified against a calf thymus DNA standard (Sigma-Aldrich) as described by Rago et al. (1990). RNA content was measured using SYBR Green II RNA Gel Stain (MoBiTec) and quantified against calf liver RNA standard (Sigma-Aldrich) measured by FLx 800 Fluorescence Microplate Reader (Bio-Tek instruments) following the manufacturer’s instructions. Total protein content was determined using Pierce™ BCA Protein Assay Kit (Thermo Fisher Scientific, Inc., USA) according to the manufacturer’s instructions. The activities of creatine kinase (CK, EC 2.7.3.2), lactate dehydrogenase (LDH, EC 1.1.1.27), and NADP-dependent isocitrate dehydrogenase (ICDH, EC 1.1.1.42) were photometrically measured in Spectramax 250 Microplate Reader (Molecular Devices) using CK-NAC-Hit kit (IFCC method, BIOMED Labordiagnostik GmbH) and NADPH (Sigma-Aldrich) based on standard curves, respectively. Complete nucleic acids, protein, and enzyme analyses were performed at room temperature from supernatant of 100 mg white muscle tissue of wild (*n* = 10) and farmed (*n* = 7) pikeperch and were reported as amount per gram of white muscle tissue. The enzyme activity is given as international unit (IU) per gram muscle.

### Gene expression analyses

The total RNA of snap-frozen samples was isolated using the QIAzol® lysis reagent (Qiagen) according to the manufacturer’s instructions. To increase the quality of RNA, a purification step with the RNeasy Mini Kit (Qiagen) according to the manufacturer’s guidelines was performed. Isolated total RNA was quantified with NanoDrop ND-1000 spectrophotometer (Peqlab). The iScript cDNA Synthesis Kit (Bio-Rad) was used to reverse transcribe 1 µg RNA to cDNA. The expression was measured in duplicates of 20 ng cDNA template using the LightCycler® 96 System (Roche) and FastStart Essential DNA Green Master (Roche) according to the manufacturer’s instructions. After pre-incubation, a three-step run at 95 °C for 10 s, 60 °C for 10 s, and 72 °C for 25 s, as well as the following melting curve analysis, is performed. Primers for genes of interest were tested and described in detail by Franz et al. (2022). For normalisation, the two reference genes Elongation factor 1 alpha (*Eef1a1*) and Ribosomal protein S5 (*Rps5*) were used (Swirplies et al. 2019). Table 1 summarises the gene list of all used primers. The expression of single genes Paired box 3 (*Pax3*), Myogenic factor 5 (*Myf5*), Insulin-like growth factor 1 (*Igf1*), Insulin-like growth factor 2 (*Igf2*), and Myostatin (*Mstn*) in wild (*n* = 10) and farmed (*n* = 7) pikeperch was displayed as normalised relative quantities (NRQ) using both reference genes (Vandesompele et al. 2002).

**Table 1:** Gene list (alphabetical) with published primers used in the present study for quantitative RT-PCR with the LightCycler® 96 System (Roche)

Symbol	Accession no	Gene description	Sense 5′-3′ Antisense 5′-3′
*Eef1a*1	XM_031315932.1	Elongation factor 1-alpha 1	ATGGACAGACCCGTGAGCATG TTCTTGATGTAGGTGCTCACTTC
*Igf1*	XM_031313442.1	Insulin-like growth factor 1	TGTGTGGAGAGAGAGGCTTTTAT AGCGAGCAGCCTTGCTAGTCT
*Igf2*	XM_031282902.1	Insulin-like growth factor 2	GAGGCTTCTATTTCAGGTAGGC ACGGGTATGACCTGCAGAGAG
*Mstn*	XM_028591409.1	Myostatin	ACTGGGGCATCGAGATCAACG TTGGGGCCCTCTGAGATCTTAA
*Myf5*	HM190249.1	Myogenic factor 5	GTGGAAAACTACTACGGCCTAC TCGTTCCTCGCATATGAATAACC
*Pax3*	XM_031283761.1	Paired box 3	ACCCACGCTGGCTCAGAACTA CTCCACGATTTTATGTCGGATGT
*Rps5*	SLUC_FBN_1	Ribosomal protein S5	GCAGGATTACATTGCTGTGAAAG TCATCAGCTTCTTGCCATTGTTG

### Protein expression analyses

Protein was extracted from muscle tissue using the CelLytic™ MT cell lysis reagent (Sigma-Aldrich), including 1% Protease Inhibitor Cocktail (Sigma-Aldrich), and the Precellys homogeniser (Bertin Instruments) with 2.8 mm Zirconium oxide beads. Protein concentrations were determined using the protein A280 mode of the NanoDrop 1000 spectrophotometer (Peqlab). Protein abundances of Pax3, Myf5, Igf1, Igf2, and Myostatin were determined with a Jess Simple Western device (ProteinSimple, Bio-Techne). An overview of used antibodies and detected protein size is given in Table 2 and in Supplemental Fig. S1. Samples were diluted to 0.5 mg/ml (Pax3, Myf5, Myostatin) or 1 mg/ml (Igf1 and Igf2) according to the manufacturer’s instructions and separated with the 12–230 kDa Separation Module (ProteinSimple, Bio-Techne). All antibodies were diluted 1:20, and the RePlex feature and Total Protein Assay (ProteinSimple, Bio-Techne) were employed to determine the amount of total protein within the same capillary for normalisation. For the blocking step, 5% nonfat dry milk was used in myostatin detection and Rotiblock (Roth) instead of antibody diluent for Igf1 and Igf2. The anti-goat (Igf1) and the anti-rabbit (Pax3, Myf5, Igf2, Myostatin) Horseradish Peroxidase (HRP) detection module (ProteinSimple, Bio-Techne, article no. 043–522-2, DM-001) were employed to detect antibody binding. The data were analysed with the Compass for Simple Western Software (v.6.1.0, Build 0106, ProteinSimple, Bio-Techne). Specific binding of the antibodies was shown before (Grunow et al. 2021).

**Table 2:** Proteins and used primary antibodies for Protein abundance detection by Jess Simple Western device (ProteinSimple, Bio-Techne)

Protein, used antibody	Company	Article no	Theoretical MW^1^ [kDa]
Paired box 3, anti-PAX3 antibody	abcam	ab180754	53.1
Myogenic factor 5, anti-Myf5 antibody	abcam	ab125301	26.0
Insulin-like growth factor 1, anti- IGF1 (Internal Region) antibody	Antikörper-online	ABIN571240	20.4
Insulin-like growth factor 2, anti- IGF2 (AA 25–91) antibody	Antikörper-online	ABIN1078199	24.5
Myostatin, anti-MSTN (AA 300–350) antibody	Antikörper-online	ABIN674469	44.6

^1^ MW—molecular weight in Dalton

### Histological analyses

To obtain a complete muscle cross section, slices of caudal peduncle from pikeperch were cut in 10-µm-thick serial transversal sections using a cryostat microtome (Leica CM 1950) at − 20 °C. Slices were stained using Haematoxylin and Eosin (H&E) as well as NADH-diaphorase staining as routine procedures (Harris 1900, Novicoff et al. 1961). The staining incubation at 37 °C of Harris haematoxylin (3 min) and eosin (0.1%, 5 min) was adapted to the pikeperch muscle sample. In addition, for NADH diaphorase staining, the formol-calcium fixation (4%, 45 s) was shortened, and the incubation time for tetrazolium salt medium was extended to 75 min. Muscle cross sections were viewed with an Olympus BX43 microscope, and images were taken with the UC30 camera (OSIS) using 100 × and 200 × magnification. At least three images were analysed using the interpolating polygon function of the interactive measurement module of the software Cell^F (OSIS) by measuring 300 randomly selected fibres per region. Cell nuclei were counted in H&E-stained sections of the white muscle (1.76 ± 0.02 mm^2^ area) and the red muscle (0.72 ± 0.07 mm^2^ area) and extrapolated to the total area size.

### Immunohistochemical analyses

The immunohistochemical staining of fish tissue sections followed the procedure described for two salmonid species by Grunow et al. (2021). The 10-µm cross sections of wild (*n* = 10) and RAS-farmed (*n* = 5) pikeperch samples were fixated (4% PFA, 10 min), washed (1 × PBS, 2 × 5 min), and permeabilised (5% BSA with 0.5% TritonX-100, 1 h) at room temperature. The primary antibodies, rabbit polyclonal anti-Pax3 antibody (1:400, abcam ab180754) or rabbit polyclonal anti-Myf5 antibody (1:200, Abcam ab125301), were incubated for 2 h at room temperature and then kept at 2–8 °C overnight. As secondary antibodies Alexa Flour 594 goat anti rabbit IgG H&L (1:500, Abcam ab150080) and Alexa Flour 488 goat anti rabbit IgG H&L (1:500, abcam ab150077) were used. The incubation time was 1.5 h. After washing (1 × PBS, 2 × 5 min), sections were mounted with Roti Fluor Care DAPI (Roth) and were imaged using a Leica DM400B fluorescence microscope in combination with a Leica DFC320 camera at 100 × magnification. Images of fluorescence-labelled nuclei were analysed with Cell^F image analysis software (OSIS). Immunohistochemically stained nuclei were counted in 1.0–1.8 mm^2^ of the red region and 2.1–2.8 mm^2^ of the white region.

### Statistical and data analyses

Data are presented as mean ± SEM (Standard error of the mean). For data of biochemical and expression analysis, the comparison of the two origins of pikeperch was performed using a two-tailed Student’s *t* test of SAS software version 9.4 (Statistical Analysis Institute Inc., USA) with a significance level of *p* < 0.05. For multiple comparisons in muscle structure analyses of wild and RAS-farmed pikeperch, a general linear model (GLM procedure, SAS 9.4) was used. The GLM for the muscle fibre structure analysis included main effects for origin (one degree of freedom (DF)) and muscle fibre region (two DF), as well as their interaction term (two DF). The error term had 39 DF. In the GLM for the nuclei analysis, the DF for the origin, muscle fibre region, and interaction terms exhibited one DF, respectively, and the error term had 26 DF. Pearson correlation analyses of all parameters measured were carried out to detect any correlations between the multivariate parameters and were executed by SAS software version 9.4 (Statistical Analysis Institute Inc., USA). The significance level *p* < 0.05 of the main effect means, distinguishing group effects (wild versus RAS-farmed pikeperch, independent from muscle type), muscle effects (white, intermediate, red muscle independent from group) and the group-muscle interaction.

## Results

### Amount of nucleic acid, protein content, and enzyme activity

**Fig. 2:**
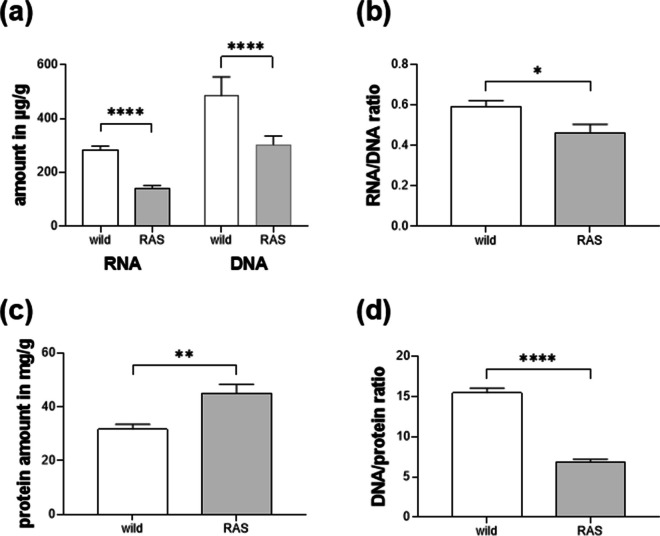
Biochemical characteristics of white muscle tissue in pikeperch of the two origins Lake Hohen Sprenz (wild, *n* = 10, white column bar) and farmed in RAS (RAS, *n* = 7, grey column bar). **a** Total amount of RNA and DNA in µg/g muscle, **b** resulting ratio indices RNA/DNA, **c** amount of protein in mg/g muscle, and **d** index DNA/protein. Statistical analyses were performed by two-tailed Student’s *t* test (SAS 9.4). *p < 0.05, **p < 0.01, ****p < 0.0001

The white muscle tissue differed between wild pikeperch and RAS-farmed pikeperch in terms of protein synthesis capacity. The RNA and DNA content of the muscle was higher in wild pikeperch than in RAS pikeperch (Fig. 2a; *p* < 0.0001). In consequence, wild animals had a RNA/DNA ratio of 0.59 and differed from RAS animals (Fig. 2b; *p* < 0.05). Although the protein content of muscle in RAS pikeperch was 30% higher than that in wild pikeperch (Fig. 2c), the DNA/protein ratio was 1.25 times higher in the wild than in the farmed fish (Fig. 2d; *p* < 0.0001).

**Fig. 3:**
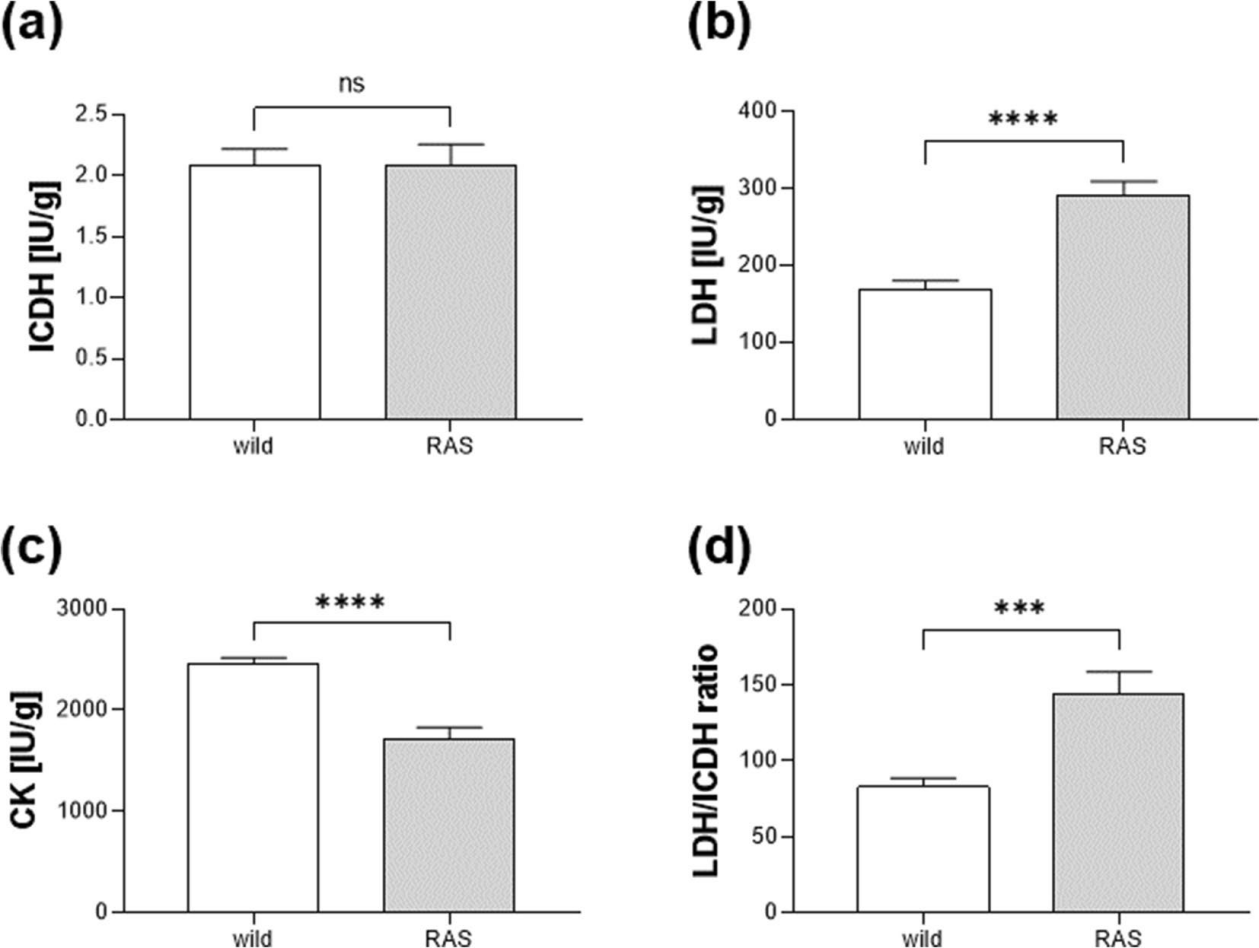
Enzyme activities of **a** isocitrate dehydrogenase (ICDH), **b** lactate dehydrogenase (LDH), and **c** creatine kinase (CK) in IU per gram muscle tissue and **d** resulting LDH/ICDH ratio of lake Hohen Sprenz pikeperch (wild, *n* = 10, white column bar) and RAS-farmed pikeperch (RAS, *n* = 7, grey column bar). Statistical analyses were performed by two-tailed Student’s *t* test (SAS 9.4). ns—not significant, ***p < 0.001, ****p < 0.0001

Analysis of the enzymatic activity showed no differences in ICDH levels between wild and cultured animals (Fig. 3a). In contrast, the LDH and CK levels were different in white muscle. The LDH activity of the Lake Hohen Sprenz animals was 1.7 times lower and the CK activity 1.4 times higher compared to the RAS animals (Fig. 3b, c, *p* < 0.0001). Consequently, the calculated LDH/ICDH ratio in the white muscle was 83 in wild pikeperch and 144 in RAS-farmed pikeperch (Fig. 3d, *p* = 0.0004).

### Muscle specific gene and protein expression

**Fig. 4:**
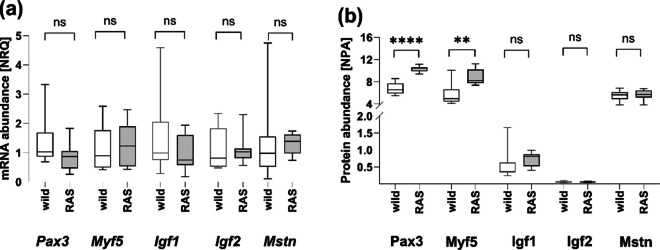
Muscle specific expression on mRNA (**a**) and protein (**b**) level in white muscle of pikeperch of the two origins lake Hohen Sprenz (wild, *n* = 10, white box plot) and cultured in RAS (RAS, *n* = 7, grey box plot). Results are shown as normalised relative quantity (NRQ) to reference genes Elongation factor 1-alpha 1 (*Eef1a1*) and Ribosomal protein S5 (*Rps5*) as well as normalised protein abundance (NPA). Statistical analyses were performed by two-tailed Student’s *t* test with a significance level of *p* < 0.05 (SAS 9.4). ns—not significant, ** *p* < 0.01, **** *p* < 0.0001

The expression of muscle-specific genes in the fast white muscle tissue showed no differences between the wild and the RAS-farmed pikeperch. Transcription factors involved in stem cell activation and myoblast formation, represented here by *Pax3* and *Myf5,* were expressed similarly in wild and cultured pikeperch (Fig. 4a). Genes associated with myotube formation, the subsequent stage in the muscle development cascade, including insulin-like growth factors *Igf1* and *Igf2*, exhibited similar expression levels in both pikeperch origins (Fig. 4a). Similarly, myofibre formation and size regulator *Mstn* showed comparable expression level in animals of both origins (Fig. 4a). In contrast, differences in muscle-specific protein abundance were present depending on the origin of the fish. The RAS-farmed pikeperch showed higher protein abundance of Pax3 and Myf5 in contrast to wild pikeperch (Fig. 4b, *p* < 0.0001 respectively *p* = 0.0031). The protein abundance of Igf1, Igf2, and Mstn was similar in wild and RAS-farmed pikeperch muscle tissue (Fig. 4b).

### Muscle fibre and nuclei density in pikeperch

**Fig. 5:**
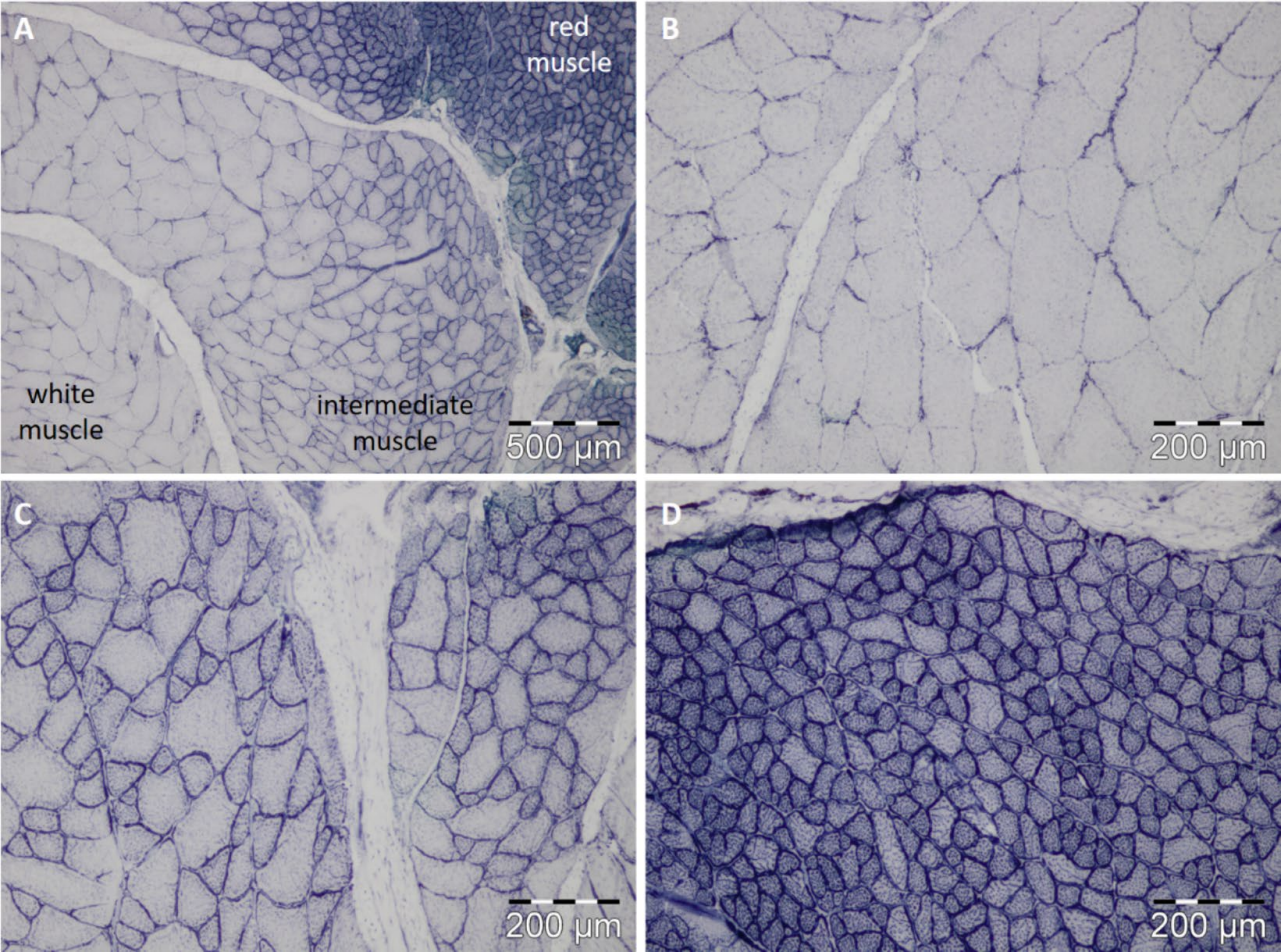
Transverse muscle cross-sectional area of caudal peduncle of *S. lucioperca* (**A**) with detailed view of white (**B**), intermediate (**C**), and red (**D**) muscle regions displayed by NADH-diaphorase staining

Three different muscle regions (white, intermediate, and red) were distinguishable in the muscle cross section of pikeperch (Fig. 5). In general, the muscle fibre structure of pikeperch differed between the white, intermediate, and red muscle but not between the two origins wild pikeperch and RAS-farmed pikeperch. In both origins, the average fibre area in white muscle was around 3.6 and 7.2 times higher than in intermediate and red muscle, respectively (muscle effect *p* < 0.0001, Table 3). In the wild and RAS pikeperch, the average muscle fibre diameter was around 110 µm in white muscle, 60 µm in intermediate muscle, and 40 µm in red muscle (muscle effect, *p* = 0.0004, Table 3).

**Table 3:** Muscle fibre structure of *S. lucioperca* analysed by image analysis in NADH-diaphorase stained muscle cross sections. Results are given as mean ± SEM of wild pikeperch from lake Hohen Sprenz (*n* = 10) and pikeperch from recirculating aquaculture system (RAS, *n* = 5), separated into white muscle (white), intermediate muscle (inter), and red muscle (red). Significant differences (*p* < 0.05) of group effects (wild vs. RAS), muscle effects (white, intermediate, red muscle), and the interaction of group and muscle were calculated by general linear model (GLM procedure, SAS 9.4)

Parameter	Wild pikeperch			RAS-farmed pikeperch			Group effect	Muscle effect	Group × Muscle
	White	Inter	Red	White	Inter	Red	*p*	*p*	*p*
Fibre area (µm^2^)	12,096.8 ± 577.7	3483.3 ± 302.4	1674.9 ± 54.4	11,602.5 ± 750.6	3064.9 ± 411.5	1628.3 ± 25.2	0.063	< 0.0001	**0.245**
Average diameter (µm)	110.9 ± 2.6	59.9 ± 2.6	43.8 ± 0.7	110.7 ± 4.1	56.4 ± 3.6	43.8 ± 0.2	0.013	0.000	**0.491**
Smallest diameter (µm)	37.2 ± 2.0	22.4 ± 1.5	19.5 ± 0.4	28.1 ± 1.6	20.7 ± 1.5	17.9 ± 0.9	0.121	0.029	**0.985**
Largest diameter (µm)	249.8 ± 13.8	154.9 ± 5.3	76.9 ± 2.4	251.1 ± 6.1	154.8 ± 12.2	86.8 ± 3.8	0.009	< 0.0001	**0.652**
% diameter < 20 µm	Not detected	0.1 ± 0.1	0.5 ± 0.2	Not detected	0.9 ± 0.4	1.4 ± 0.5	0.002	0.002	**0.062**
% diameter 20–50 µm	3.8 ± 0.9	39.4 ± 4.5	69.6 ± 4.2	7.7 ± 2.6	46.8 ± 4.9	67.0 ± 3.7	0.014	< 0.0001	**0.437**
% diameter > 50 µm	96.2 ± 0.9	60.5 ± 4.5	29.9 ± 4.2	92.3 ± 2.6	52.4 ± 5.2	31.6 ± 3.5	0.044	0.001	**0.524**

The intermediate and red muscle had a similar smallest fibre diameter of around 20 µm in contrast to two times larger cells in white muscle (*p* = 0.029, Table 3). The diameter of the largest fibres was also different between the three muscle regions (*p* < 0.0001, Table 3). In animals of both origins, the maximum fibre size of 250 µm was detected in white muscle. In the intermediate muscle of adult pikeperch, the maximum fibre size was about 100 µm and in the red muscle 170 µm smaller, than in the white muscle (Table 3).

Fibres with a diameter of < 20 µm were in negligible proportion in intermediate and red muscle and non-existent in white muscle of wild and RAS-farmed pikeperch (Table 3). In contrast, the proportion of fibres of 20–50 µm changed in the three muscle regions (muscle effect, *p* < 0.0001, Table 3). This size class accounted for two thirds of the red muscle fibre and nearly half of the intermediate region. In the white region, the proportion of this fibre size corresponded to only 4%, respectively 8% (Table 3). The proportion of > 50 µm fibres in the three regions behaved in the opposite way to the 20–50 µm fibres. In the white muscle, more than 90% of the fibres were larger than 50 µm, in the intermediate muscle around 50 to 60%, and in the red muscle only around 30% of the cells were in this size range (muscle effect, *p* = 0.001, Table 3).

Nuclei were analysed in total areas of 1.76 ± 0.02 mm^2^ in the white muscle and 0.72 ± 0.07 mm^2^ in the red muscle. The nuclei density per mm^2^ was 3.5 to 4 times higher in red muscle than in white muscle independent of the origin of the fish (Table 4). However, the number of nuclei per fibre was higher in the white muscle than in the red muscle with no differences between the fish origins (Table 4). The same applied to the myonuclear domain (ratio µm^2^ fibre area/nucleus). In the white muscle, the myonuclear domain was 3.2 respective 4.5 times higher compared to the red muscle (Table 4). In both, the white and red muscle, the wild pikeperch had significantly lower values for this parameter (*p* < 0.0001, Table 4).

**Table 4:** Nuclei in white and red muscle of *S. lucioperca* using cross sections with H&E staining. Results are given as mean ± SEM of wild pikeperch from lake Hohen Sprenz (*n* = 10) and pikeperch from recirculating aquaculture (RAS, *n* = 5), separated into white muscle (white), and red muscle (red). Significant differences (*p* < 0.05) in group effects (wild vs. RAS), muscle effects (white vs. red), and the interaction of group and muscle were calculated by general linear model (GLM procedure, SAS 9.4)

Nuclei	Wild pikeperch		RAS-farmed pikeperch		Group effect	Muscle effect	Group × Muscle
	White	Red	White	Red	*p*	*p*	*p*
Nuclei density of 1 mm^2^	587.87 ± 17.15	1898.94 ± 67.38	222.30 ± 34.90	961.39 ± 144.80	0.439	0.001	**0.855**
Nuclei per fibre	7.10 ± 0.32	3.17 ± 0.15	2.48 ± 0.26	1.55 ± 0.21	0.180	0.027	**0.402**
Myonuclear domain	1707.66 ± 51.77	529.84 ± 18.96	5072.79 ± 935.74	1133.31 ± 156.12	< 0.0001	< 0.0001	**0.000**

### Abundance of myogenic precursor cells

Immunohistochemistry was applied to identify the number of myogenic precursor cells (Pax3 and Myf5 positive cells, Supplemental Fig. S2 and Fig. S3) in the white and red muscles of the two origins. As already shown in Table 4, the white and red muscles had significantly different numbers of nuclei per mm^2^, but the proportion of Pax3 and Myf5 positive progenitor cells was similar (Table 5). Considering the white and red muscles, the white fibres of the RAS-cultured pikeperch had a higher proportion of myogenic Pax3 stem cells compared to the wild pikeperch (*p* = 0.034, Table 5). Furthermore, a higher number of Myf5 positive cells was detected in red muscle compared to white muscle (*p* = 0.016, Table 5) in RAS-farmed pikeperch. Consequently, the proportion of Myf5 positive nuclei in the red muscle was twice as high as in the white muscle (Table 5). In contrast to the farmed fish, wild pikeperch showed a similar number and distribution of Myf5-positive nuclei in white and red muscle regions (Table 5).

**Table 5:** Quantification of Pax3 and Myf5 positive cells analysed in muscle cross sections *S. lucioperca* of two origins using immunohistochemistry and image analysis. Results are given as mean ± SEM of wild pikeperch from lake Hohen Sprenz (*n* = 10) and pikeperch from recirculating aquaculture (RAS, *n* = 5), separated into white muscle (white) and red muscle (red). Significant differences (*p* < 0.05) in group effects (wild vs. RAS), muscle effects (white vs. red), and the interaction of group and muscle were calculated by general linear model (GLM procedure, SAS 9.4)

Parameter	Wild pikeperch		RAS-farmed pikeperch		Group effect	Muscle effect	Group × Muscle
	White	Red	White	Red	*p*	*p*	*p*
number Pax3 +	8.00 ± 0.89	6.00 ± 1.44	11.40 ± 4.50	5.40 ± 1.17	0.088	0.168	**0.211**
% Pax3 +	0.31 ± 0.03	0.25 ± 0.06	0.80 ± 0.3	0.27 ± 0.07	0.007	0.099	**0.034**
number Myf5 +	154.55 ± 11.66	104.99 ± 18.03	38.20 ± 5.91	128.40 ± 20.22	0.882	0.547	**0.016**

### Multivariate correlation analysis

**Fig. 6:**
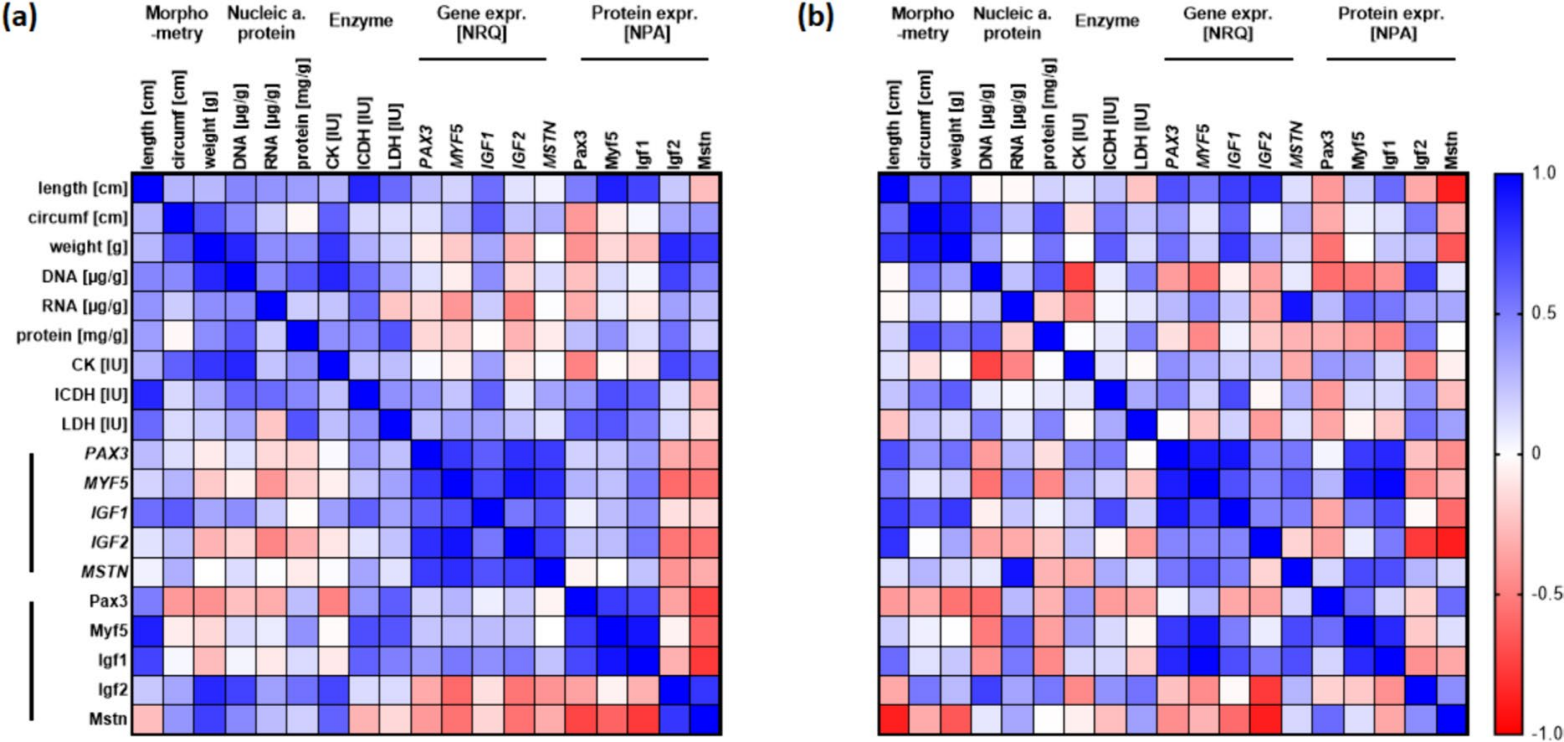
Multivariate correlation matrix of morphological, enzymatic, genetic, and protein parameters of muscle tissue distinguished between **a** wild and **b** farmed pikeperch. The Pearson correlation coefficients have been assigned colour codes to indicate their direction and strength (white—neutral coefficients, red—negative coefficients, blue—positive coefficients)

Multivariate correlation analysis was used to analyse and illustrate the relationships among the various data. The study revealed that in wild pikeperch there are predominantly positive correlations between morphometric and biochemical traits (nucleic acids and protein content, enzyme activities). Furthermore, genetic factors and their related protein abundance in the muscle show a clear clustering (Fig. 6a). As expected, transcription factors of muscle development were positively correlated with each other. Protein abundance followed the gene expression relationships with exception of Igf2 and Mstn, which showed a negative correlation to the three remaining transcription factors. The desired homology in the morphometry of the RAS-farmed pikeperch, as evidenced by a higher positive correlation between the morphometric values, leads to changes in the correlations with the other parameters (Fig. 6b). The distinct clustering pattern of the parameters was eliminated, and altered correlations were observed among the examined muscle biological factors. As demonstrated in Fig. 6, the correlation to individual parameters was reversed in wild and RAS-farmed fish. For example, the creatine kinase activity of RAS fish was negatively associated with the RNA and DNA content of the muscle, in contrast to the positive correlation in wild pikeperch.

## Discussion

The primary objective of this study was to investigate and compare the histological structures, enzymatic activity, gene expression, and protein abundance of pikeperch muscle from a natural lake or from RAS. Hereby, this fundamental study aims to provide objective comparison standards that allow to improve product quality in the pikeperch aquaculture industry in the long term. The present study included a multivariate analysis of various muscle parameters. A clustering of these parameters potentially indicates underlying connections in the muscle development, their function, as well as alterations induced by aquaculture conditions in pikeperch. Some of the positive correlations between parameters present in wild pikeperch are missing in RAS-farmed fish, which could indicate a systematic change originating from their aquaculture background.

### Amount of nucleic acid, protein content, and enzyme activity in pikeperch

A commonly used biochemical performance parameter in fish muscle is the RNA/DNA ratio. The ratio is based on a variable RNA content, which depends on the nutritional status as well as the age of the fish, and a relatively constant DNA content (Bastrop et al. 1992; Buckley et al. 1999; Bulow 1987; Meyer et al. 2012). Studies have shown that the variation in DNA content between different fish was lower than expected (Horstkotte and Rehbein 2006). The RNA content of muscle is more variable depending on growth, condition, stress, and nutritional status (Hussna et al. 2020). In our study, the RNA and DNA content in wild pikeperch was higher compared to RAS fish. A higher RNA content indicates higher protein synthesis, making the RNA/DNA ratio a valuable indicator of growth rate and overall condition (Bulow 1987). Previous investigations focusing on the RNA/DNA ratio, primarily conducted on larvae and juveniles (Bastrop et al. 1992, Buckley et al. 1999, Ferron and Leggett 1994, Suthers et al. 1996, Tielmann et al. 2017), consistently revealed that well-fed, actively moving young fish display a higher RNA/DNA ratio compared to starved, lethargic, and metabolically inactive individuals. In our study, RAS-farmed pikeperch showed higher protein content, indicating a faster growth rate compared to wild pikeperch. In contrast, the higher RNA/DNA ratio observed in the white muscle of wild pikeperch indicates a greater potential for protein synthesis. However, this potential for protein synthesis is not utilised in the wild pikeperch studied. The mathematical quotient of DNA and protein indicates a lower efficiency in protein synthesis. This raises the question of whether the low protein synthesis in wild pikeperch is due to a lack of necessity or ability to synthesise more protein. Nonetheless, the potential for higher protein synthesis, indicated by elevated RNA and DNA content, is present. These findings support the hypothesis that wild pikeperch is more metabolically active than RAS-farmed pikeperch.

To gain insights into the metabolic cycle, we investigated muscle-specific enzyme activities involved in muscle growth. Hereby, white and red muscle differs in fibre types in both mammals and fish (Gagaoua et al. 2016, Gil et al. 2003, Johnston et al. 1977, Wieser et al. 1987). LDH activity serves as a marker for the characterisation of energy metabolism and has been extensively studied in fish muscle from the 1960s to 1980s (Bouck and Ball 1968, Frankel 1980, Lim et al. 1975, Wilson et al. 1973). This end product of anaerobic glycolysis varies among species and is affected by temperature, pressure, salinity, feed, and swimming activity (Sullivan and Somero 1980, Storey 2016). Our study showed origin-specific differences in pikeperch, with higher LDH values observed in RAS-farmed individuals. We hypothesised that the elevated water temperature in RAS systems contributes to the increased LDH content in the muscles of farmed fish. Another important metabolic parameter is the enzyme CK. Investigations involving 15 fish species have demonstrated that fishes generally have a higher creatine content in muscle compared to mammals (Hunter 1929). Creatine plays a vital role in providing continuous energy to the skeletal muscle and has been used as a dietary supplement to enhance muscle growth (Aziza et al. 2020; Mabrouk et al. 2020; Adeshina and Abdel-Tawwab 2021). However, while there have been positive effects of creatine supplementation in African catfish (*Clarias gariepinus*) and Nile tilapia (*Oreochromis niloticus*), the muscle CK content and growth performance of gilthead seabream (*Sparatus aurata*, Schrama et al. 2018, Ramos-Pinto et al. 2019) and European seabass (*Dicentrarchus labrax*, Schrama et al. 2022) were not significantly affected. In our study, the CK content was higher in wild pikeperch compared to RAS-farmed animals, suggesting a greater muscular energy demand in wild and more active fish. The ICDH content describes the oxidative capacity of muscle (Holloszy et al. 1970; Dohm et al. 1973). Pikeperch exhibited 2.4 to 3.6 times lower ICDH content in muscle compared to salmonids such as maraena whitefish (*Coregonus maraena*) and rainbow trout (Grunow et al. 2021), regardless of their origin or husbandry. These species-specific differences may be attributed to distinctions, with pikeperch, typically categorised as standers and salmonids as swimmers, as well as variances in their diet (Barroso et al. 1994; Rufino-Palomares et al. 2016).

### Muscle specific gene expression on mRNA and protein level

Generally, insights into changes in gene expression profiles are valuable, particularly for designing feeding programs in pikeperch aquaculture or understanding the impact of climate change on wild pikeperch populations. Prior studies examining gene expression in pikeperch have primarily focused on embryonic and larval development (Schäfer et al. 2021; Venuto et al. 2020; Żarski et al. 2020a; Franz et al. 2022), as well as on aspects related to reproduction and stress (Hermelink et al. 2011, Swirplies et al. 2019, Żarski et al. 2020b). First attempts of comparative transcriptome studies in pikeperch looked for evolutionary differences between percid species (Xie et al. 2019), or carried out transcriptome assemblies for multiple tissues (Nguinkal et al. 2021). However, in the current study, no differences were found in the muscle-specific gene expression between wild and farmed pikeperch. Nevertheless, complex interactions between gene expression and muscle DNA content exist (Adams and Haddad 1996; Horstkotte and Rehbein 2006). The significance of these interactions is exemplarily shown by the insulin-like growth factor (IGF) system, the main actor of muscle growth in all vertebrates (Johnston et al. 2011, Reinecke et al. 2005). Despite this understanding, many questions remain unanswered regarding the signalling pathways for *Igf1* expression in fish (Fuentes et al. 2013). Whether there are relationships between mRNA expression, DNA, and protein content in the fish muscle remains to be investigated, especially in pikeperch with its lifelong growth.

Proteomic analyses reveal changes in protein identities, abundances, and post-translational modifications and have been used to study patterns of protein expression in fish concerning various factors such as development, diet, or environmental influences (Bradley et al. 2002, Ghaedi et al. 2016, Hogstrand et al. 2002, Islam 2006, Martin et al. 2003). In general, fishes are able to recruit new myocytes via the transcription factor Myf5, enabling them to continuously form muscle fibre throughout their lives (Johansen and Overturf 2005; Koumans et al. 1991). Additionally, the muscle satellite cell marker Pax3 is also involved in muscle development, growth, repair, and lifelong, indeterminate growth (Froehlich et al. 2013, Kassar-Duchossoy et al. 2005, Relaix et al. 2005). Comparative protein data of wild and RAS-farmed pikeperch revealed higher Myf5 and Pax3 protein abundances in RAS-farmed pikeperch. This indicates that farmed pikeperch have better conditions for muscle growth, likely due to increased food availability. The differences observed between mRNA and protein expression underscores the need to combine both approaches to understand fish biology.

### Myofibre and nuclei in pikeperch

The presence of muscle fibres of various sizes characterises mosaic hyperplasia, which is normally restricted to embryonic and larval development (Rescan 2008; Johnston et al. 2011). Here, we detected different myofibre sizes in the white, intermediate, and red regions in the muscle cross section of adult pikeperch of both origins. The maximal diameter of white muscle, reaching around 250 µm, falls within the species-specific functional maximum of 100–300 µm, indicating hypertrophic growth potential as suggested by Rowlerson and Vegetti (2001). Smaller fibres (< 20 µm) were absent in the white muscles and accounted for less than 1% in the intermediate and red muscles. On average, the fibres in the intermediate and red muscles were about half the size of those in the white muscles. Altogether, this indicates a more hypertrophic growth of the white muscle in the adult pikeperch. Although there are no or few small muscle fibres (< 20 µm), the increased protein abundance of Myf5 and Pax3 in RAS-farmed pikeperch indicates improved conditions for muscle growth. To understand muscle growth in more detail, comparable studies in other size classes of pikeperch still have to be implemented. The impact of starvation, compensatory growth, and nutrition or summer–winter differences on fibre size represent promising fields for further investigations. Besides these histological parameters, the amount and origin of myonuclei are important for muscle biology, since it determines the content of DNA for gene transcription (Allen et al. 1999). The data presented here show the fibre-specific number of nuclei in pikeperch, with higher nucleus density in red muscle and thus higher protein turnover rate in red muscle (Tseng et al. 1994). The origin of the pikeperch did not influence these parameters. Furthermore, the size of the myonuclear domain, i.e., the theoretical volume of cytoplasm associated with a single myonucleus, correlates with muscle fibre type and fibre size (Jimenez and Kinsey 2012). In hypertrophy–induced muscle fibre growth of fish, the myonuclear domain size increases and is associated with higher DNA content (Jimenez and Kinsey 2012). In comparison to studies on maraena whitefish and rainbow trout, the number of nuclei per fibre is similar in pikeperch, but the myonuclear domain is almost twice as large in the white and red muscle of pikeperch as in the salmonids (Grunow et al. 2021). This is consistent with the larger average fibre size measured in pikeperch. Additionally, farmed pikeperch showed higher levels of myonuclear domain compared to wild animals. This suggests pronounced hypertrophic muscle growth and potentially larger fibre size in RAS-grown pikeperch with increasing size of this lifelong, indeterminate growing fish.

## Conclusion

Overall, the muscle of wild pikeperch appears to exhibit a higher metabolic activity compared to RAS-farmed pikeperch. Wild pikeperch are exposed to varying environmental parameters such as water temperature, photoperiod, spawning season, feed composition and intake, and swimming behaviour, all well-known influencers of fish muscle physiology. In contrast, aquaculture practices offer the advantage of controlling and stabilising these environmental factors, aiming to optimise the growth and overall health of farmed pikeperch for economic purposes. In regards to the muscle growth and biology of fish, several gaps and key questions remain. Similar to most fishes, pikeperch exhibit indeterminate growth throughout their lives. Combined with their rapid growth, early maturation, breeding performance, and desirable fillet flavour, pikeperch are promising candidates for RAS, as they exhibit higher growth potential under these optimal conditions. To improve our understanding of the general muscle characteristics of this freshwater species, we provide detailed information on the wild-caught pikeperch and factors influencing muscle development and function. Together with improvements in pikeperch husbandry, the increasing knowledge of pikeperch muscle tissue is crucial for animal-specific adaptation of aquaculture facilities and the production of a high fillet quality for consumers. This has implications for understanding the mechanisms underlying patterns and relationships in muscle biology.

### Supplementary Information

Below is the link to the electronic supplementary material.

Supplementary file1 (TIF 2.12 MB)

Supplementary file2 (TIF 1.68 MB)

Supplementary file3 (TIF 1.75 MB)

## Data Availability

All relevant data are within the manuscript.
