# Supplementary figures and images for: Pikeperch muscle tissues: a comparative study of structure, enzymes, genes, and proteins in wild and farmed fish
Source: Fish Physiol Biochem. 2024 May 11;50(4):1527–44. doi: 10.1007/s10695-024-01354-1 (PMC11286731; doi:10.1007/s10695-024-01354-1)

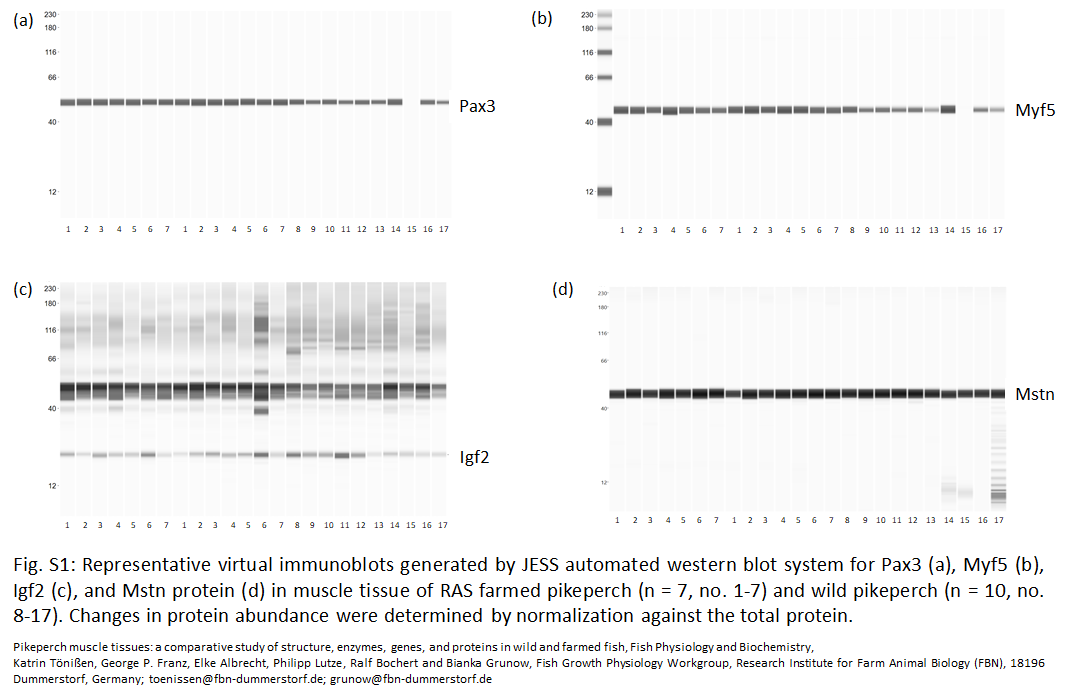

Supplement: Supplementary file 1 — Supplementary file1 (TIF 2.12 MB) [file 10695_2024_1354_MOESM1_ESM.tif]

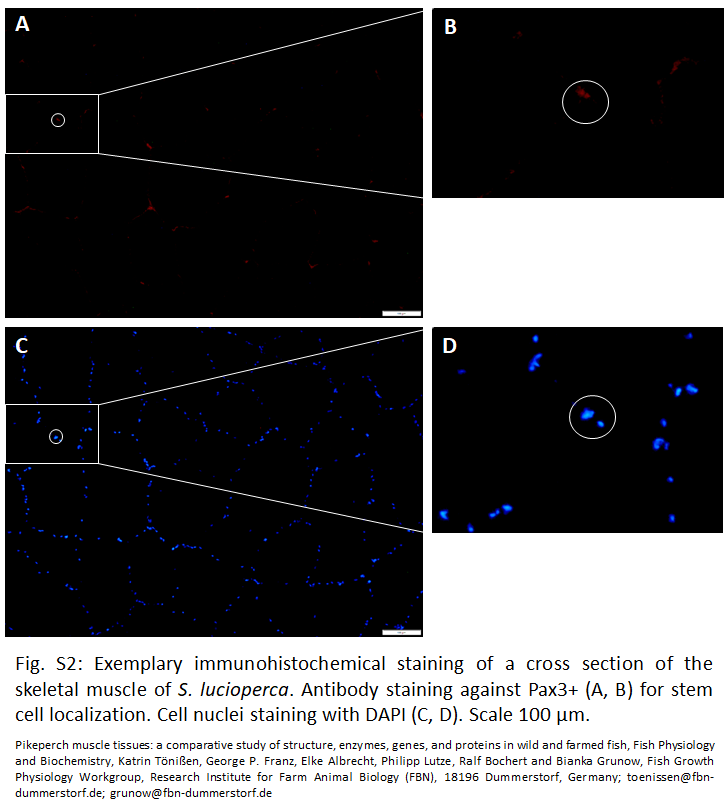

Supplement: Supplementary file 2 — Supplementary file2 (TIF 1.68 MB) [file 10695_2024_1354_MOESM2_ESM.tif]

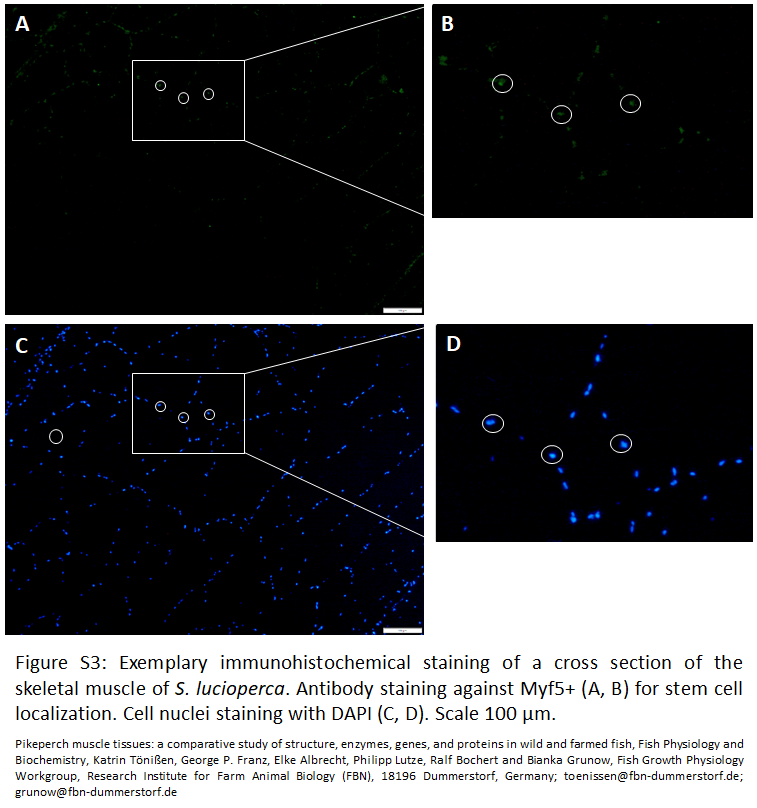

Supplement: Supplementary file 3 — Supplementary file3 (TIF 1.75 MB) [file 10695_2024_1354_MOESM3_ESM.tif]
